# Do Personally Tailored Videos in a Web-Based Physical Activity Intervention Lead to Higher Attention and Recall? – An Eye-Tracking Study

**DOI:** 10.3389/fpubh.2014.00013

**Published:** 2014-02-12

**Authors:** Stephanie Alley, Cally Jennings, Nayadin Persaud, Ronald C. Plotnikoff, Mike Horsley, Corneel Vandelanotte

**Affiliations:** ^1^Centre for Physical Activity Studies, Institute for Health and Social Science Research, Central Queensland University, Rockhampton, QLD, Australia; ^2^Faculty of Physical Education and Recreation, University of Alberta, Edmonton, AB, Canada; ^3^Learning and Teaching Education Research Centre, Central Queensland University, Noosa, QLD, Australia; ^4^Priority Research Centre for Physical Activity and Nutrition, University of Newcastle, Newcastle, NSW, Australia

**Keywords:** physical activity, health promotion, web-based, eye-tracking, tailoring

## Abstract

Over half of the Australian population does not meet physical activity guidelines and has an increased risk of chronic disease. Web-based physical activity interventions have the potential to reach large numbers of the population at low-cost, however issues have been identified with usage and participant retention. Personalized (computer-tailored) physical activity advice delivered through video has the potential to address low engagement, however it is unclear whether it is more effective in engaging participants when compared to text-delivered personalized advice. This study compared the attention and recall outcomes of tailored physical activity advice in video- vs. text-format. Participants (*n* = 41) were randomly assigned to receive either video- or text-tailored feedback with identical content. Outcome measures included attention to the feedback, measured through advanced eye-tracking technology (TobiiX 120), and recall of the advice, measured through a post intervention interview. Between group ANOVA’s, Mann–Whitney *U* tests and chi square analyses were applied. Participants in the video-group displayed greater attention to the physical activity feedback in terms of gaze-duration on the feedback (7.7 vs. 3.6 min, *p* < 001), total fixation-duration on the feedback (6.0 vs. 3.3 min, *p* < 001), and focusing on feedback (6.8 vs. 3.5 min, *p* < 001). Despite both groups having the same ability to navigate through the feedback, the video-group completed a significantly (*p* < 0.001) higher percentage of feedback sections (95%) compared to the text-group (66%). The main messages were recalled in both groups, but many details were forgotten. No significant between group differences were found for message recall. These results suggest that video-tailored feedback leads to greater attention compared to text-tailored feedback. More research is needed to determine how message recall can be improved, and whether video-tailored advice can lead to greater health behavior change.

## Introduction

Physical activity improves physical and mental health, and significantly lowers the risk of non-communicable disease including cardiovascular disease, diabetes mellitus, and cancer (1). It is estimated that individuals who are physically active have a 30–50% lower risk of non-communicable diseases and a 20–50% lower risk of mortality than inactive individuals (2–4). The World Health Organisation recommends 30 min of moderate intensity activity on 5 days of the week to receive health benefits and reduce the risk of non-communicable disease (5). Despite this, more than 50% of Australians fail to meet these recommendations (6), which is estimated to cost the Australian economy 13.8 billion each year in healthcare, loss of productivity, and mortality costs (7). Hence, there is an urgent need for effective physical activity interventions with a broad reach.

Innovative web-based physical activity interventions have been developed to take advantage of the high percentage of Australians (79%) with access to the Internet in their homes (8). Not only do health interventions delivered via the Internet have the potential to reach a large audience at low-cost, they are convenient for the participants and enable the content to be delivered in a non-confrontational way (9–11). Although the short-term effectiveness of web-based physical activity interventions is well established, participant retention and engagement have been identified as a challenge with many web-based interventions reporting high dropout rates or low use of the websites (12, 13). As exposure to the intervention content is strongly linked to behavioral outcomes, low participant retention and engagement may be limiting the effectiveness of the web-based interventions (14, 15).

Web-based health interventions that provide personalized advice improve engagement and behavioral outcomes compared to interventions that provide generic advice (16, 17). Computer-tailored advice is the personalized feedback that is automatically produced using a computer-based expert system that delivers feedback based on participant’s responses to a questionnaire (17). Computer-tailored feedback is commonly delivered in text-based format on intervention websites, despite users tending to skim and scan text on the Internet rather than engage in concentrated reading (18). The use of rich media content, including graphics and videos, has become very common on the Internet, and users have become accustomed to this (19). Furthermore information presented in video-format has been found to result in improved recall of website content (20, 21), improved engagement, and to facilitate a stronger emotional response than text in educational settings (22, 23). Information presented through videos in web-based health interventions may therefore be an effective way of engaging users and be more effective in producing behavior changes.

To date, only a small number of web-based health interventions have used videos to deliver program content, and only one provided video with personalized content to participants (24, 25). Vandelanotte and colleagues (24) developed and conducted the pilot testing of a physical activity intervention with two modules of either text- or video-tailored feedback. Video-group participants received their activity feedback in video-format with a presenter and animated graphical images, whilst text-group participants received their feedback in text-format, which included static graphics. Results demonstrated that inactive participants who received computer-tailored physical activity feedback in video-format had greater improvements in physical activity levels than participants receiving traditional computer-tailored feedback in text-based format with identical content; however a more conservative intention-to-treat analysis found no significant differences between the two groups (26). A study conducted by Lee (27) found that participants in a web-based health intervention, which delivered content through videos had greater levels of self-reported attention, interactivity, overall website evaluation and preference than participants assigned to a static intervention site. These results suggest that videos may be more effective at engaging participants in web-based tailored health information, and have the potential to improve the behavioral outcomes of text-tailored feedback. Further research is required to understand how participants process video- and text-delivered information and to determine whether video-tailored physical activity feedback leads to greater observed engagement, understanding, and recall than traditional text-tailored physical activity feedback.

Eye-tracking technology can be used to objectively measure participant’s attention and engagement in web-based health interventions. Eye-tracking technology has been used in marketing and educational research to record users eye-gazes on web-delivered information (28, 29). The eye-tracking data provide a physiological measure that is directly linked to the cognitive processing (30), and has been beneficial in understanding how people attend to and process information on a web-page (28). Past health studies have used eye-tracking data to determine what types of health promotion advertisements attract attention. These studies also found that eye-gaze predicted correct recall of the advertisements, demonstrating the importance of attention for learning (30, 31). To our knowledge no web-based physical activity interventions have used eye-tracking technology to understand the way users interact with and attend to personal activity information. Eye-tracking technology can therefore improve our understanding of how user’s process health advice delivered through text and video on the Internet. Such findings will enable health promotion workers and researchers to make adjustments to the delivery of information on web-based behavior change interventions and improve participant’s engagement in the intervention content.

The aim of this study was to examine, with the use of an eye-tracking device and a recall questionnaire, the differences between video-tailored and text-tailored physical activity feedback in terms of participant attention to and recall of the intervention content. It was hypothesized that participants receiving video-tailored feedback would spend more time paying attention to the feedback, be less distracted and have improved recall of their personal physical activity feedback in comparison to those receiving text-tailored feedback.

## Materials and Methods

### Procedure

A two group randomized trial was conducted to compare participant’s processing of text- and video-tailored physical activity feedback. Participants were recruited via e-mail from staff and students of Central Queensland University Noosa campus. To be eligible participants had to be English speaking, be over 18 years of age and be familiar in using the Internet for general purposes. Data were collected from each participant in one 20–25-min session from March to June 2012. To begin the session, participants were seated at a computer in a quiet research room where they were un-interrupted. Participant’s eyes were calibrated with an eye-tracking device connected to the computer. Participants were then invited to complete a demographic questionnaire on the computer. Next participants were provided with access to the “My Physical Activity Advice” website where they completed two modules of tailored feedback in either video- or text-format. The intervention website automatically assigned participants at random to receive either text- or video-tailored physical activity feedback as they signed into the website. The researcher supervising the session was unaware of the randomization sequence. While participants were completing the intervention, the eye-tracking device video recorded and produced data of their eye movements. After completing the intervention the researcher asked participants nine brief questions to test their recall of the intervention content. Ethical clearance was obtained from the Central Queensland University Human Ethics Committee (project number H13/04-044).

### Intervention

The two module web-based physical activity intervention with video- and text-tailored advice was previously developed by Vandelanotte and colleagues (24). The intervention has been found to be effective at increasing participant’s physical activity levels (26). The content and structure of the text-based and video-based feedback was identical, only the method of delivery was different. The computer-tailored content was tailored to participant’s physical activity levels, as assessed by the “Active Australia Questionnaire (AAQ)” (32), participant demographics (age, body mass index (BMI), work environment, and the distance to often-visited places) and psychosocial correlates of physical activity that were based on the theory of planned behavior (attitudes, subjective norm, perceived behavioral control, and intention) (33). The intervention provided normative feedback by comparing participant’s physical activity to the minimum and optimal physical activity guidelines in a bar graph. Participant’s perceived benefits and barriers to becoming more active were also discussed. The intervention consisted of two modules. Participants can receive up to 7 sections of feedback in the first module, which focuses on the benefits of physical activity, and up to 10 sections of feedback in the second module, which focuses on creating an active lifestyle. A more detailed description of the intervention can be found elsewhere (26).

### Measures

#### Demographics

The pre-test demographic survey collected information on: gender, age, height and weight (to calculate BMI), highest level of education, current employment status, household income level, and motivation to increase physical activity through the question “do you want to increase your physical activity?” with two response options, yes and no.

#### Attention

Participant’s visual attention to the personalized video- and text-tailored feedback was measured with a TobiiX 120 eye-tracking device. The TobiiX 120 tracks eye movements at a resolution of 1,280 pixels and at a controller refresh rate of 60–75 Hz. It allows 15° of head movement 60 cm from the screen (Tobii Technology AB, 2008). Eye-gazes on the screen including fixation, when participant’s eye-gaze focuses on one point, and saccades, when participant’s eye-gaze moves from one fixation to fixate on another point, were recorded at 15 ms. The eye-tracking software, Tobii studio can be set to record fixations and saccades in a selected area of interest. The area on the computer screen in which the feedback was displayed (the video or text) was chosen as an area of interest. Tobii studio software calculated data on gaze-duration (the total time of both fixations and saccades), and fixation-duration (the total time of all fixations) for the area of interest as well as the total computer screen (Tobii Technology AB, 2008). Gaze-duration in areas on the screen outside the area of interest was calculated as a measure of distraction. The video recording of participant’s eye movements was used to measure the focusing-duration, by measuring the duration of actually reading by text-group participants or watching key parts of the video (e.g., presenter, graph) by video-group participants. Due to the potential measurement error when recording focusing-duration, a second researcher re-timed the focusing-duration of 10 (24%) randomly selected participants to test the inter-researcher reliability. The video was also used to record the number of feedback sections participants skip before they have finished reading or watching the advice in full. Gaze-duration outside the feedback area of interest was recorded as a measure of distraction. The proportion of gaze-duration in the feedback area compared to gaze-duration in the entire screen and the proportion of fixations compared to gaze-duration in the feedback area were also calculated as measures of distraction.

#### Recall

The post intervention recall interview was conducted immediately after each participant received their physical activity feedback. The interview consisted of nine open-ended questions. The questions assessed participant’s understanding of the goal of the feedback they received, their memory of the feedback they received (including the recommendations for physical activity, their own physical activity levels, and the benefits of physical activity) and their understanding of the graph comparing their physical activity levels to the recommendations. The interview duration was approximately 5 min. Participant responses were recorded using an audio digital recorder (Livescribe Pen), and transcribed in Microsoft Word. Each question was coded as correct or incorrect. A total recall score was also calculated for each participant by summing the total number of correct recall responses the participant gave on all questions. Possible scores ranged from 0 to 9.

### Analysis

#### Data screening

All analyses were conducted using SPSS version 19. Significance level was set at *p* < 0.05. Descriptive statistics were calculated for participant demographic information. A chi square analysis was conducted to compare group baseline participant characteristics. All continuous variables were screened for outliers and normality using Fisher’s skewness coefficient. The proportion of time participants spent viewing the feedback compared to the entire screen, and the number of feedback sections skipped were found to have a significantly skewed distribution. Square root, logarithm, and inverse transformations were unsuccessful to transform these variables. Therefore Mann–Whitney *U* tests were used to analyze the data from these variables.

#### Attention

A series of four one-way between groups Analyses of Variance (ANOVA’s) were conducted to compare video- and text-participants on attention, which included gaze-duration, fixation-duration, and focusing-duration in the feedback area, and number of sections skipped. Bonferroni correction was applied to control for the risk of a false positive arising from the four comparisons of attention and group. A *p* value score of *p* < 0.01 was therefore required for any of the attention and group analyses to be deemed significant. Three Analyses of Variance were also conducted to compare video- and text-participants on distraction (gaze-duration in the areas outside the feedback), the proportion of gaze-duration spent in the feedback area compared to other areas on the screen, and the proportion of fixation-duration compared to gaze-duration. Bonferroni correction was applied to control for the risk of a false positive arising from the three comparisons of distraction and group. A *p* value score of *p* < 0.017 was therefore required for any of the distraction and group analyses to be deemed significant. The number of feedback sections participants read or watched was entered as a covariate in all attention analyses.

#### Recall

A chi square analysis was conducted to determine whether there was a between group (video and text) difference in the mean number of correct responses to each question. An Analysis of Variance was conducted to compare the total recall scores in video- and text-participants. Next, the total recall score was dichotomized using a median split in order to examine the relationship between group, recall, and attention. An Analysis of Variance was conducted to compare gaze-duration in the feedback area and recall (high total recall score vs. low total recall score) with the covariates group (video vs. text), and number of feedback sections.

## Results

The demographic details of the participants are documented in Table 1 below. Data were collected from 41 participants. Participants were randomly assigned to the video- (*n* = 21) or text-group (*n* = 20). There were no baseline differences between the two intervention groups for participant characteristics.

**Table 1:** **Participant demographics for total group and for video- and text-groups**.

		Total *n* (%)	Video *n* (%)	Text *n* (%)
		(*n* = 41)	(*n* = 21)	(*n* = 20)
Gender	Males	14 (34)	7 (33)	7 (35)
	Females	27 (66)	14 (67)	13 (65)
Age	18–30	12 (29)	8 (38)	4 (20)
	31–50	15 (37)	5 (24)	10 (50)
	>50	14 (33)	8 (38)	6 (30)
BMI	Normal (<25)	23 (57)	11 (55)	12 (60)
	Overweight (≥25)	17 (43)	9 (45)	8 (40)
Motivation	Motivated	31 (76)	16 (76)	15 (75)
	Not motivated	10 (24)	5 (24)	5 (25)
Education	Secondary school	9 (22)	5 (24)	4 (20)
	TAFE	7 (17)	3 (14)	4 (20)
	University	25 (61)	13 (62)	12 (60)
Employment	Full time	13 (32)	7 (33)	6 (30)
	Part time	17 (42)	8 (38)	9 (45)
	Unemployed	3 (7)	1 (5)	2 (10)
	Student	8 (20)	5 (24)	3 (15)
Income	<30,000	16 (39)	9 (43)	7 (35)
	30,001–70,000	11 (26)	5 (24)	6 (30)
	>70,001	14 (35)	7 (33)	7 (35)

### Attention

**Figure 1:**
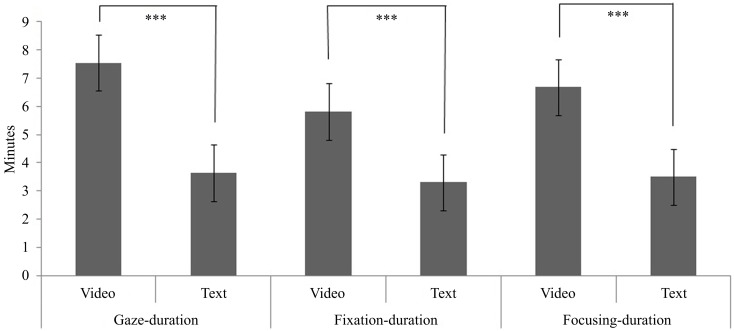
**Gaze-, fixation-, and focusing-duration in the feedback area by group (video, *n* = 20; text, *n* = 17)**.

As shown in Figure 1 and Table 2, Gaze-duration within the feedback area was significantly higher in the video-group than the text-group, *F*(1, 36) = 30.39, *p* < 001. Furthermore, video-group participants had a significantly greater fixation-duration and focusing-duration, *F*(1, 36) = 13.09, *p* < 001; *F*(1, 36) = 20.85, *p* < 001 respectively. The inter-researcher reliability of the focusing-duration variable was very high, as indicated by a Krippendorff’s alpha of 0.99. Researcher 1 timed the 10 participants who were measured by both researchers to have a mean of 4.8 (SD = 2.9) minutes focusing on the feedback and researcher 2 timed these participants to have a mean of 4.9 (SD = 3.0) minutes focusing on the feedback. Video-group participants finished 95% of their feedback sections (*M* = 0.75, SD = 2.2) compared to the text-group participants who finished only 66% of their feedback sections (*M* = 4.6, SD = 3.9), this difference was significant at *p* < 0.001.

**Table 2:** **Descriptive statistics for gaze-, fixation-, and focusing-duration in the feedback area and distraction by group (video and text)**.

		Attention						Distraction	
		Gaze-duration feedback area (min)		Fixation-duration feedback area (min)		Focusing-duration on feedback (min)		Gaze-duration other areas on screen (min)	
Group	*n^a^*	*M* ± SD	*F*	*M* ± SD	*F*	*M* ± SD	*F*	*M* ± SD	*F*
Video	20	7.72 ± 2.00	30.39***	5.96 ± 1.93	13.09***	6.82 ± 1.94	20.85***	1.56 ± 0.68	29.33***
Text	17	3.63 ± 2.43		3.30 ± 2.19		3.50 ± 2.38		0.42 ± 0.56	

***p < 0.001.

*Adjusted for number of feedback sections*.

*^a^Eye-tracking data was missing from one video and three text-participants*.

As seen in Table 2, distraction, measured by the length of gaze-duration within areas on the screen other than the feedback area was significantly higher in video- compared to text-group participants *F*(1, 36) = 29.33, *p* < 0.001. The proportion of gaze-duration within the feedback area compared to gaze-duration within the total screen was significantly lower in video-group participants (*M* = 82%, SD = 8.83%) than text-group participants (*M* = 87%, SD = 16.15%; *p* < 0.01). The proportion of time participants spent fixating on the feedback from the total time they spent viewing the feedback was 76.28% (SD = 11.07) in the video-group and 83.27% (SD = 9.02) in the text-group. This difference was not significant *F*(1, 36) = 4.01, *p* = 0.053.

### Recall

The percentage of correct responses for each of the recall interview questions for total group and the video- and text-groups are presented in Table 3. A chi square analysis revealed that there were no between group recall differences for any of the questions.

**Table 3:** **Correct responses for each recall question by group and chi square comparison of correct responses in video- and text-groups**.

	Correct response			Chi square χ^2^
	Total group *n* (%)	Video-group *n* (%)	Text-group *n* (%)	
Q1. What is the goal of the advice?	33 (80)	15 (71)	18 (90)	2.25 ns.
Q2. What is the recommended amount of physical activity?	25 (61)	12 (57)	13 (65)	0.27 ns.
Q3. What is the optimal amount of physical activity per day?	21 (51)	11 (52)	10 (50)	0.02 ns.
Q4. Are you meeting the physical activity guidelines?	36 (88)	18 (86)	18 (90)	0.18 ns.
Q5. Exactly how many minutes of physical activity do you do on a weekly basis?	35 (85)	17 (81)	18 (90)	0.67 ns.
Q6. What was presented in the graph	14 (34)	7 (35)	7 (33)	0.01 ns.
Q7. What was each of the bars in the graph showing?	15 (37)	8 (38)	7 (35)	0.04 ns.
Q8. How will meeting the physical activity recommendations benefit you?	34 (83)	17 (81)	17 (85)	0.12 ns.
Q9. What chronic diseases can be prevented?	33 (80)	18 (86)	15 (75)	0.75 ns.

*ns., not significant*.

The mean total recall response was 5.86 (SD = 2.26) in the video-group and 6.15 (*SD* = 1.63) in the text-group. No significant relationship between total recall and group (video vs. text) was found *F*(1, 36) = 0.22, *p* = 0.639. Based on the total recall scores 17 participants were assigned to the low recall category, and 21 to the high recall category using a median split. The mean gaze-duration in the feedback area for participants with high recall was 5.51 (SD = 2.83) minutes, and 6.28 (SD = 3.20) minutes for participants with a low recall score. No significant relationship was found between attention and recall.

## Discussion

The findings demonstrate that video-tailored advice is more effective at gaining participant’s visual attention than text-tailored advice in a web-based physical activity intervention. Video-group participants spent significantly longer viewing their feedback, had a higher sum of fixations on the feedback, and spent longer focused on the key parts of the feedback than text-group participants. Furthermore, video-group participants finished a significantly greater amount of feedback sections than the text-group participants despite both groups having the ability to navigate through their feedback and finish sections prematurely. The objective eye-tracking data confirms the findings from Lee (27) of improved self-reported attention to video-presented health information compared to identical text-presented information. The findings also demonstrate that the improved engagement toward video-messages compared to text-messages observed in marketing and educational settings applies in a web-based health behavior intervention setting (22, 23). The finding of improved attention in video-group participants is important for the development of web-based health interventions as high exposure to the intervention content is associated with improved behavior change (14, 15). Presenting health advice through video may be an effective strategy to improve the low levels of participant engagement and therefore exposure to web-based health interventions.

There might be several reasons to explain the improved attention to the message in the video-group participants. Firstly, the improved attention in the video-group participants could in-part be a result of participant’s expectations. Website users have come to expect interactive websites with rich media content due to the current Internet environment where popular websites are employing rich media content to engage users (19). Secondly, the higher attention in video-group participants could be due to participant’s social and emotional connection to the feedback. The presenter delivering the feedback and the images of active people in the video may produce a greater emotional and social connection to the feedback. This is in line with previous research that found students to have a greater emotional response to information delivered in video compared to text (23). Thirdly, the improved attention in video-group participants could be due to the lower level of mental effort required from the video-group participants. Text requires users to actively read in order to comprehend the text, whilst watching a video requires a lower mental effort (34). This might also be the reason why text-group participants skipped more feedback sections than video-group participants. Lastly, the higher level of attention seen in the video-group participants may be due to the perceived control video- and text-group participant’s have to move through their feedback. Although both video- and text-group participants were able to click over to the next section of the feedback at any time, text-group participants more frequently clicked over to the next section before they had finished their current section. Internet-users typically have less control over the pace of information they receive through video compared to text (35), and could therefore be in the habit of watching videos to the end, without navigating through them. Furthermore, text-group participants were forced to click to read the next section, whereas transitions through the video sections were automatic. As such video-group participants may have been less aware of their ability to fast-forward through the video-feedback.

Although video-group participants spend longer viewing their feedback than the text-group participants, they demonstrated greater levels of distraction than text-group participants. Video-group participants spend significantly longer viewing other areas on the screen whilst the feedback was being presented and viewed the feedback area on the computer screen for a significantly lower percentage of time than text-group participants. This may also have resulted from the difference between groups in perceived control to move through the feedback faster (35). It is likely that text-group participants clicked through to the next section immediately when they chose not to read any more of the section they were on. Whereas video-group participants tended to continue watching each section until it finished. It is likely that video-group participants were looking outside of the feedback area when they had brief moments of distraction or when they perceived some sections of the feedback as less interesting. Alternatively, they could have been listening to the audio of the video without paying close attention to what was on the computer screen. The measure of attention produced by the eye-tracking device is based upon visual attention only, and does not account for the audio of the video. Whilst it is important to note the increased distraction in the video-group participants, it is more important that video-group participants spent longer viewing the feedback, as they were less likely to skip to the next section when momentarily distracted.

Although video lead to a higher level of attention, no group differences were found for the number of correct responses on each of the recall questions, or the total number of correct responses for all of the recall questions. This is incongruent with past research findings of video leading to a greater recall in marketing and education settings (20, 21). Furthermore, the lack of relationship between attention and recall was not expected as past research demonstrated a significant positive relationship between attention and recall (30, 31). It is possible the questionnaire used did not adequately measure recall, as many participants might have had prior knowledge of some answers such as their level of physical activity, the benefits of physical activity, and the diseases associated with inactivity. Furthermore, participants in both groups had a very high number of correct responses, which may have been due to the interview being conducted immediately after the intervention. This might have created a ceiling effect where the low variability in participant’s responses made it difficult for any group differences to be detected. If there was a greater time gap between the intervention and the recall questionnaire group differences resulting from participant’s attention to the feedback may have been detected. Further research is needed to determine whether increased attention to video leads to greater recall and behavior changes, with the use of a pre-post test design, and a comprehensive recall questionnaire conducted with a longer time gap after the intervention to adequately assess recall.

Finally, the recall questionnaire outcomes revealed that participants remembered the main messages of the advice very well, but the details were much less well retained. The majority (at least 80%) of participants knew what the goal of the advice was, could remember if they were meeting the guidelines or not, knew how many minutes of physical activity they did on a weekly basis, could list how physical activity could benefit them and could recall the diseases physical activity helps to prevent. However less than half of participants could recall that the recommendations and their own activity levels were presented in the physical activity graph, and just over half of participants could correctly recall the minimum and optimal physical activity recommendations.

### Limitations

Although eye-tracking technology has improved our understanding of how to engage participants in online health interventions, the nature of the eye-tracking data poses some limitations. The eye-tracking technology only measures visual attention, not auditory attention. Furthermore, it is possible that the longer gaze- and fixation-duration in the video-group were because it took longer to watch the videos than it would take to read the same information in text-format. Another limitation with the eye-tracking technology is the use of eye-gaze as an attention measure. It is possible that participants were looking within the feedback area but are not actually processing the feedback, however given the outcomes on focusing-duration (which was much higher in the video-group) this is doubtful. Finally, there may have been error in the measurement of BMI due to the use of self-reported data.

## Conclusion

The findings support the hypothesis that video-delivered content is an effective way of improving participant’s attention to tailored health information in a web-based physical activity intervention. This is important for the development of future web-based physical activity interventions as attention and engagement are strongly linked with behavioral outcomes. Future research is required to evaluate the effectiveness of video-tailored advice in producing long-term behavior changes in comparison to standard text-tailored advice. Furthermore, future research with a larger sample size is needed to conduct analyses on the two-way interactions between participant demographics (gender, BMI, motivation to become more active, and age) and group (video and text) on attention. It is, for example, important to determine whether personalized video content is effective at increasing activity levels in older or overweight participants, as they are at higher risk for developing chronic diseases. The findings did not support the hypothesis that video-group participants would have a higher recall of the intervention content. Due to the high percentage of participants in both groups with correct responses to many of the questions, further research with more sensitive measures is needed to confirm this finding. However, the low levels of recall, especially for the physical activity recommendations highlight a need for future research to evaluate ways of improving recall of the key parts of physical activity advice. Overall, this research using eye-tracking data demonstrates that video-tailored advice leads to a higher level of attention compared to text-tailored advice in a web-based physical activity intervention.

## Conflict of Interest Statement

The authors declare that the research was conducted in the absence of any commercial or financial relationships that could be construed as a potential conflict of interest.
